# ﻿Microsatellite repeat mapping shows inner chromosomal diversification in highly conserved karyotypes of Asian cyprinid fishes

**DOI:** 10.3897/compcytogen.19.141557

**Published:** 2025-03-31

**Authors:** Phichaya Buasriyot, Weerayuth Supiwong, Nawarat Muanglen, Nattasuda Donbundit, Sukhonthip Ditcharoen, Phonluang Chumpol, Pasakorn Saenjundaeng, Sampan Tongnunui, Sathit Arunsang, Weera Thongnetr, Sitthisak Juntharat, Kriengkrai Seetapan, Thomas Liehr, Marcelo B. Cioffi, Petr Rab, Alongklod Tanomtong

**Affiliations:** 1 Department of Biology, Faculty of Science, Khon Kaen University, Muang, Khon Kaen 40002, Thailand; 2 Faculty of Interdisciplinary Studies, Nong Khai Campus, Khon Kaen University, Muang, Nong Khai 43000, Thailand; 3 Department of Fisheries, Faculty of Agricultural Technology, Sakon Nakhon Rajabhat University, Sakon Nakhon 47000, Thailand; 4 Department of Conservation Biology, Mahidol University, Kanchanaburi Campus, Sai Yok, Kanchanaburi 71150, Thailand; 5 Department of Program in Animal Science, Faculty of Agricultural Technology and Agro-industry, Rajamangala University of Technology Suvarnabhumi, Phra Nakhon Si Ayutthaya, Ayutthaya 13000, Thailand; 6 Division of Biology, Department of Science, Faculty of Science and Technology, Rajamangala University of Technology Krungthep, Bangkok, 10120, Thailand; 7 Department of Science, Faculty of Science and Technology, Prince of Songkla University, Pattani Campus, Mueng, Pattani 94000, Thailand; 8 School of Agriculture and Natural Resources, University of Phayao, Tumbol Maeka, Muang District, Phayao Province 56000, Thailand; 9 Institute of Human Genetics, Jena University Hospital, Friedrich Schiller University, 07747 Jena, Germany; 10 Departamento de Genética e Evolução, Universidade Federal de São Carlos, São Carlos, São Paulo, Brazil; 11 Institute of Animal Physiology and Genetics, Laboratory of Fish Genetics, Czech Academy of Sciences, Rumburska´, Liběchov, Czech Republic; † Deceased

**Keywords:** Chromosomal rearrangements, comparative cytogenetics, Family Cyprinidae, Fluorescence in situ hybridization (FISH), Microsatellites

## Abstract

The barbels of the subfamilies ´Poropuntinae´ and Smiliogastrinae within the family Cyprinidae play a significant role as a food source for fish in artisanal fisheries and are highly valued as ornamental fish in Thailand. In this study, we employed both conventional and molecular cytogenetics to analyze the karyotype of 15 fish species from two cyprinid lineages. All analyzed species had a diploid chromosome number of 2n = 50. Despite sharing the same 2n, our analyses revealed species-specific distribution patterns of the mapped microsatellite motifs [(CA)₁₅, (TA)₁₅, (CAC)₁₀, and (CGG)₁₀]. They were predominantly found at telomeric sites of all-to-few chromosomes. Additionally, some species exhibited a widespread distribution of the mapped microsatellites across the chromosomes while others showed no signal. These variations reflect the evolutionary divergence and chromosomal diversity within the cyprinids. Thus, our findings support the 2n stability in cyprinoid lineages while emphasizing the intrachromosomal evolutionary diversity accompanied by species-specific microsatellite distribution.

## ﻿Introduction

Thailand is home to around 10% of the global freshwater fish species, with at least 858 species belonging to 81 families identified in the country (Kang et al. 2009). The abundant variety of aquatic life in this region serves as a significant indication of its status as a worldwide hub for diverse species of freshwater fishes (Myers 2000). The cyprinoid lineages and specifically the family Cyprinidae (as defined by Tan and Armbruster in 2018), represent the most species-rich group of freshwater fishes. Tan and Armbruster (2018) provided a comprehensive review of the phylogenetic classification of cyprinid fishes, identifying 11 distinct subfamilies, i.e. slightly differing from the present categorization in the Catalog of Fishes (divided into 10 subfamilies; Fricke et al. 2025). Fishes belonging to the subfamilies ´Poropuntinae´ and Smiliogastrinae are of great economic importance in Thailand. They are highly valued for artisanal fishing, ornamental fish trade, and are extensively cultivated in aquaculture. According to Phimphan et al. (2020), around 100 species of ornamental fishes can be traced back to Thailand. However, despite this vast diversity, there is a significant gap in our understanding of their genetic data, including a lack of comprehensive cytogenetic examination of many species.

Chromosomal studies have significant implications for studying evolution, phylogenetics, systematics, taxonomy, and genetic diversity (e.g., Kushwaha et al. 2021; Supiwong et al. 2021; Yeesin et al. 2021; Mingkwan et al. 2023; Prazdnikov 2023). Karyotype analyses play a crucial role in fish breeding by enabling genetic control, facilitating the fast development of inbred lines, and aiding in the study of evolution (Ganai et al. 2011; Maneechot et al. 2016). Various researchers, including Chaiyasan et al. (2018, 2020), Phimphan et al. (2020), Khensuwan et al. (2023a, 2023b, 2024), and Buasriyot et al. (2024), have conducted cytogenetic studies on cyprinid fishes in Thailand. The current cytogenetic data in cyprinids show considerable variation in their diploid chromosome number (2n) among species, ranging from 42 in *Acheilognathusgracilis* Nichols, 1926 (Acheilognathidae) (Hong and Zhou 1985) to 446 in *Diptychusdipogon* Regan, 1905 (Cyprinidae) (Yu and Yu 1990). However, 2n = 50 is the predominant chromosome count, representing a fundamental, plesiomorphic pattern for the group (Sola and Gornung 2001; Knehsuwan et al. 2024). The evolution of karyotypes in Cyprinidae is marked by multiple independent polyploidization events across various species, which have contributed significantly to the observed 2n variation (Yang et al., 2015). Additionally, differentiated sex chromosomes are rare in this family, with only few reported cases of ZZ/ZW sex chromosomes, particularly in *Squaliuscarolitertii* Doadrio, 1988, *S.pyrenaicus* Günther, 1868 (Collares-Pereira et al. 1998) and *S.recurvirostris* Özuluğ et Freyhof, 2011 (Doori and Arslan 2022). A recent study integrating molecular and conventional cytogenetics also demonstrated that the sister tribe Labeonini possesses a conserved 2n and intrachromosomal rearrangements. Nevertheless, there is a scarcity of research that has employed molecular cytogenetic methods in these species. While most research relied on conventional approaches to establish the 2n and karyotype composition, recent studies have shifted their attention toward the repetitive DNA fraction, specifically microsatellite repeats (Phimphan et al. 2020; Saenjundaeng et al. 2020; Khensuwan et al. 2023, 2024).

Microsatellites are short repetitive DNA sequences that can range in length from one to six nucleotides, found in the genomes of all eukaryotic species, including those of fish species (Messier et al. 1996; Lopez-Flores and Garrido Ramos 2012). Regarding chromosomal distribution, microsatellites exhibit various patterns after physical mapping. Some are dispersed throughout the chromosomes, while others form discrete banding patterns (Ditcharoen et al. 2020; Haerter et al. 2023). For instance, in the rock bream (*Oplegnathusfasciatus* Temminck et Schlegel, 1844), certain microsatellites display distinct banding, whereas others are more widely dispersed along the chromosomes (Xu et al. 2013). Microsatellites employed as genetic markers are generally seen as evolving neutrally. Their frequency and distribution should therefore reflect the essential mutation process (Ellegren 2004). Additionally, the distribution pattern of their location on chromosomes can be specific to a particular species or similar in closely related species groups (e.g. Machado et al. 2020). Analyzing the distribution of microsatellite repeats enhances the understanding of chromosomal organization and evolution. For example, comparative chromosomal mapping of microsatellite repeats in various fish species has provided insights into their chromosomal patterns as well as potential roles in genome evolution (Ditcharoen et al. 2020). Variations in these patterns can further indicate chromosomal rearrangements, such as inversions or translocations, which are important in understanding speciation and evolutionary processes (Li et al. 2002). Microsatellites can also be used with molecular cytogenetic techniques to gather important information regarding the origins of sex chromosomes and the evolution of chromosomes (Cioffi and Bertollo 2012; Cioffi et al. 2012; Viana et al. 2022) and have garnered significant interest due to their involvement in chromosome organization, DNA recombination and replication, and gene expression (Lei et al. 2021).

In this study, we analyzed 15 cyprinid fish species from the subfamilies ´Poropuntinae´ and Smiliogastrinae using Giemsa staining and fluorescent in situ hybridization (FISH) of four distinct microsatellite motifs. This investigation provides an insight into how these repetitive DNAs are organized on chromosomes of these species and to what extent the patterns are similar or variable among them. This may constitute important information for further studies on genetic relationships, chromosomal evolution, and genetic diversity in the two cyprinid subfamilies.

## ﻿Material and methods

### ﻿Sources of individuals and chromosomal procurement

**Figure 1.**
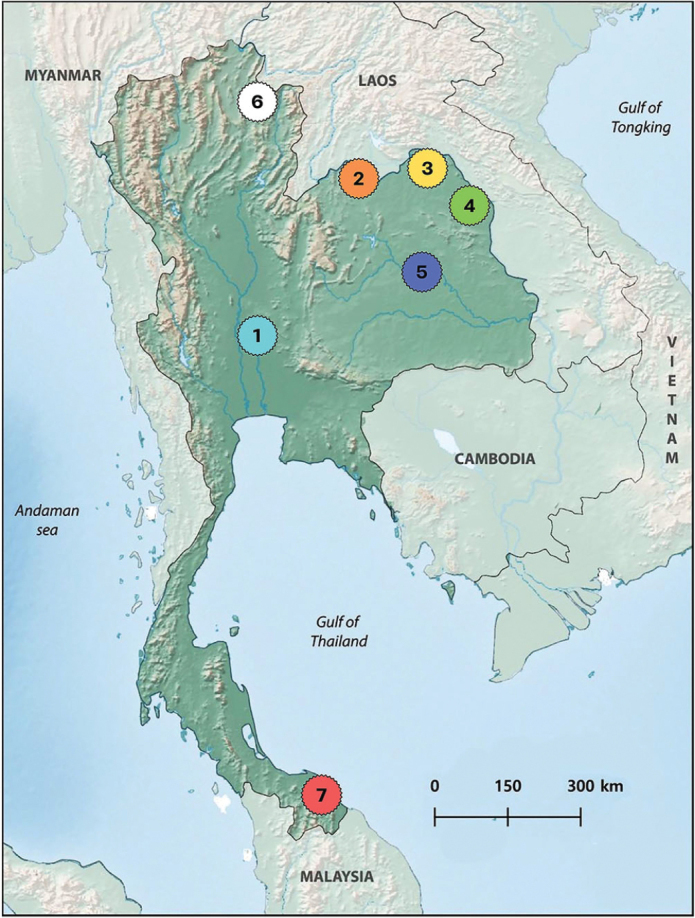
Map of Thailand showing the seven collection sites of the 15 species studied. 1. *Amblyrhynchichthysmicracanthus* (Ng et Kottelat, 2004), *Cyclocheilichthysarmatus* (Valenciennes 1842), *Cyclocheilichthysrepasson* (Bleeker, 1853), *Sikukiastejnegeri* (Smith, 1931); 2. *Cyclocheilosenoplos* (Bleeker, 1849), *Puntiusbrevis* (Bleeker, 1849), *Poropuntiuslaoensis* (Günther, 1868); 3. *Hampalamacrolepidota* (Kuhl et van Hasselt, 1823), *Barbonymusaltus* (Günther, 1868), *Barbonymusschwanenfeldii* (Bleeker, 1854); 4. *Barbonymusgonionotus* (Bleeker, 1849), *Hampaladispar* (Smith, 1934); 5. *Barbodesrhombeus* (Kottelat, 2000); 6. *Pethiastoliczkana* (Day, 1871), and 7. *Desmopuntiushexazona* (Weber et de Beaufort, 1912). The map was produced using the software QGis 3.4.4 (https://qgis.org), Inkscape 0.92 (https://inkscape.org), and Adobe Photoshop CC 2015 (San Jose, CA, USA).

Individuals of each species were collected in the river basins of Thailand (map modified from Buasriyot et al. (2024) (Fig. 1, Table 1). Live individuals were carefully transferred to the laboratory aquaria and kept in a well-aerated aquarium at 20–28 °C until analysis. To obtain the metaphase chromosomes, the specimens were treated with an intraperitoneal injection of a 0.05% aqueous colchicine solution (1 mL/100 g of body weight). They were maintained for 1 hour in a well-aerated aquarium (Bertollo et al. 2015) and sequentially euthanized using anesthetic tricaine mesylate (MS-222 euthanasia dose fish) by incorporating 25–30 mg/L of the anesthetic into the water in which the fish were immersed. Following the euthanasia of the fish, kidney tissues were excised surgically for chromosomal preparation. All procedures were approved by the Institutional Animal Care and Use Committee of Khon Kaen University, based on the Ethics of Animal Experimentation of the National Research Council of Thailand (record number IACUC-KKU-40/64), and by the RGJ committee under the number PHD/0169/2560 (Thailand).

**Table 1.** Species analyzed, collection sites and the number of analyzed individuals.

Species	Location	No. of specimens	Voucher No.*
'**Poropuntinae**'			
1. *Amblyrhynchichthysmicracanthus*	Chao Phraya River Basin, 14°51'30"N, 100°24'42"E Ton Pho, Mueang Sing Buri District, Sing Buri (site 1)	09♀; 09♂	KKU_432–449
2. *Barbonymusaltus*	Songkhram River Basin, 18°00'20.4"N, 103°28'23.6"E, So Phisai District, Bueng Kan (site 3)	11♀; 08♂	KKU_231–233, KKU_450–464
3. *Barbonymusgonionotus*	Songkhram River Basin, 17°43'12.0"N, 104°06'55.9"E, Sam Phong, Si Songkhram District, Nakhon Phanom (site 4)	10♀; 10♂	KKU_393, KKU_465–483
4. *Barbonymusschwanenfeldii*	Songkhram River Basin, 18°00'20.4"N, 103°28'23.6"E, So Phisai District, Bueng Kan, (site 3)	09♀;10♂	KKU_259–260, KKU_484–500
5. *Cyclocheilichthysarmatus*	Chao Phraya River Basin, 14°51'30"N, 100°24'42"E, Ton Pho, Mueang Sing Buri District, Sing Buri (site 1)	08♀; 11♂	KKU_501–519
6. *Cyclocheilichthysrepasson*	Chao Phraya River Basin, 14°51'30"N, 100°24'42"E, Ton Pho, Mueang Sing Buri District, Sing Buri (site 1)	07♀; 09♂	KKU_394, KKU_520–534
7. *Cyclocheilosenoplos*	Mekong River Basin, 17°52'42.0"N, 102°43'07.1"E, Mi Chai, Mueang, Nong Khai District, Nong Khai (site 2)	06♀; 12♂	KKU_535–552
8. *Poropuntiuslaoensis*	Mekong River Basin, 17°52'42.0"N, 102°43'07.1"E, Mi Chai, Mueang, Nong Khai District, Nong Khai (site 2)	10♀; 07♂	KKU_245–246, KKU_269, KKU_553–566
9. *Sikukiastejnegeri*	Chao Phraya River Basin, 14°51'30"N, 100°24'42"E, Ton Pho, Mueang Sing Buri District, Sing Buri (site 1)	08♀; 09♂	KKU_567–583
**Smiliogastrinae**			
10. *Barbodesrhombeus*	Chi River Basin, 16°13'55.2"N, 103°15'59.0"E, Tha Khon Yang, Kantharawichai District, Maha Sarakham (site 5)	07♀; 12♂	KKU_227, KKU_584–601
11. *Desmopuntiushexazona*	To Daeng Peat Swamp Forest, 6°04'31"N, 101°57'45"E, Puyo, Su-ngai Kolok District, Narathiwat (site 7)	09♀; 11♂	KKU_602–621
12. *Hampaladispar*	Songkhram River Basin, 17°43'12.0"N, 104°06'55.9"E, Sam Phong, Si Songkhram District, Nakhon Phanom (site 4)	09♀; 10♂	KKU_622–640
13. *Hampalamacrolepidota*	Songkhram River Basin, 18°00'20.4"N, 103°28'23.6"E, So Phisai District, Bueng Kan (site 3)	12♀; 07♂	KKU_247–248, KKU_270, KKU_641–656
14. *Pethiastoliczkana*	Yom River Basin, 18°54'07.0"N, 100°16'30.0"E, Chiang Muan, Chiang Muan District, Phayao (site 6)	08♀; 12♂	KKU_255–258, KKU_279–280, KKU_657–670
15. *Puntiusbrevis*	Mekong River Basin, 17°52'42.0"N, 102°43'07.1"E, Mi Chai, Mueang Nong Khai District, Nong Khai (site 2)	10♀; 09♂	KKU_402–406, KKU_671–684

* All fish samples were kept at the Vertebrate Cytogenetics Laboratory, Department of Biology, Faculty of Science, Khon Kaen University.

### ﻿Conventional analysis

Mitotic cell suspensions were dropped onto microscope slides and allowed to air-dry. The conventional staining procedure was performed using a 5% Giemsa solution in phosphate buffer (pH 6.8) for 10 minutes (Bertollo et al. 2015). The chromosomal characterization was performed using Microsoft Excel 2013 software and Adobe Photoshop CS6.5.2.3.

### ﻿Molecular cytogenetics

We mapped four microsatellites [(CA)_15_, (TA)_15_, (CAC)_10_, and (CGG)_10_] directly labeled with Cy-3 during the synthesis (Kubat et al. 2008). The hybridization procedures were performed under high stringent conditions (70%; 2.5 ng/µL probes, 2 µg/µL salmon sperm DNA, 50% deionized formamide, 10% dextran sulfate, and 2× SSC at 37 °C overnight) following the protocol described by Yano et al. (2017).

### ﻿Microscopical analysis and image processing

At least 30 metaphase spreads per individual were analyzed to confirm the 2n, karyotype structure, and results of FISH experiments. The metaphases with a clear chromosome morphology were selected, and the images captured using a Zeiss Axion Imager 7.2 epifluorescence microscope, and analyzed using Axionvision 4.8 software (Zeiss, Jena, Germany). Chromosomes were classified as metacentric (m), submetacentric (sm), subtelocentric (st), or acrocentric (a) and according to Levan et al. (1964).

## ﻿Results

**Figure 2.**
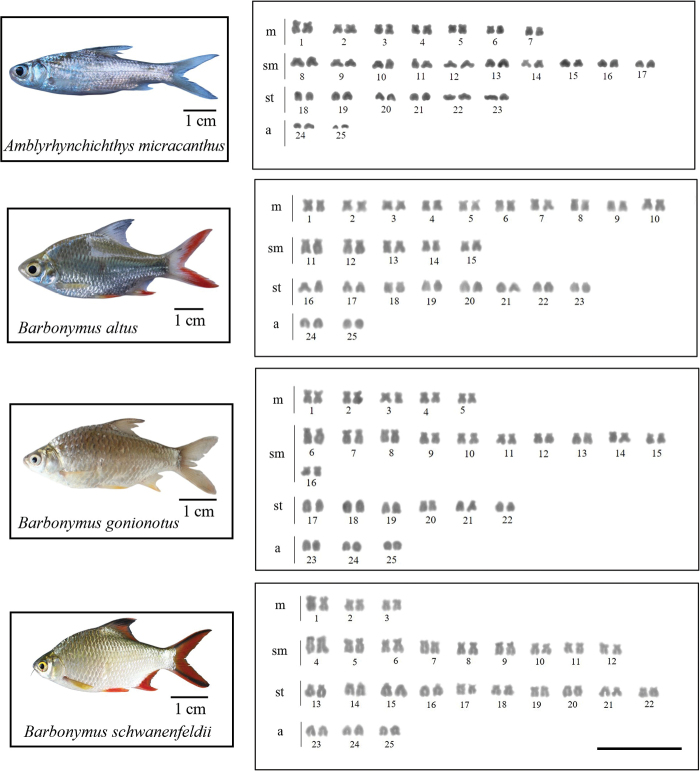
Karyotypes of four 'Poropuntinae' species arranged from Giemsa-stained chromosomes. Scale bar: 5 µm.

**Figure 3.**
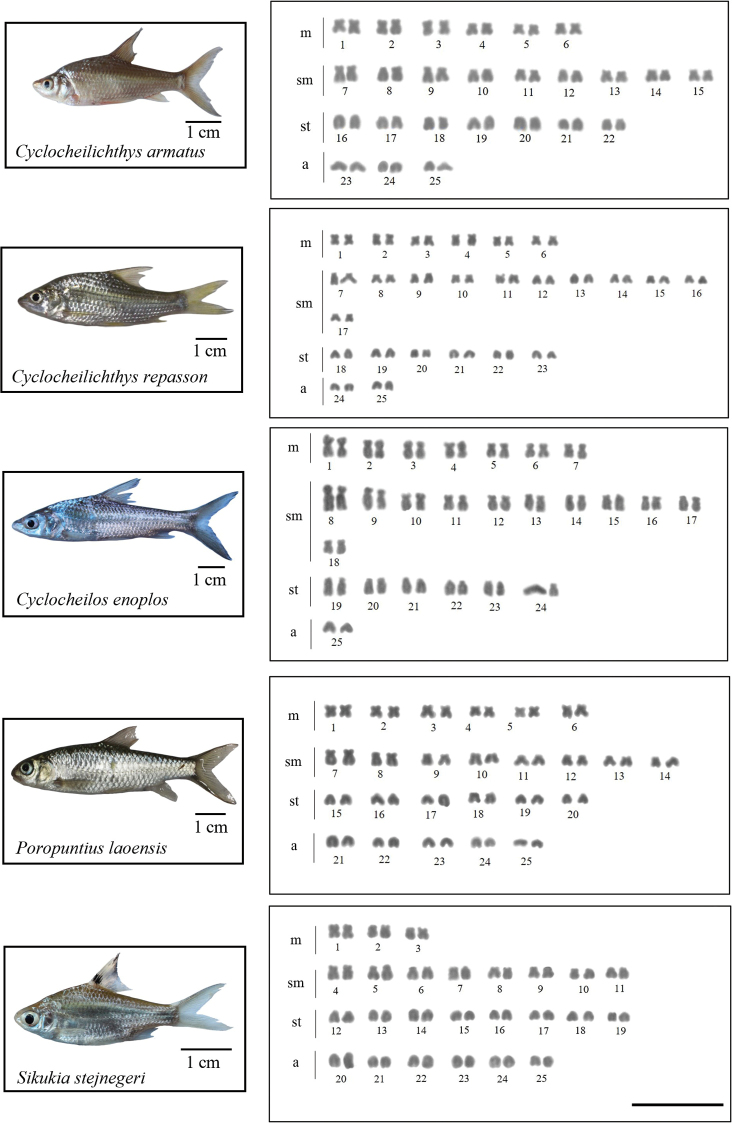
Karyotypes of five 'Poropuntinae' species arranged from Giemsa-stained chromosomes. Scale bar: 5 µm.

**Figure 4.**
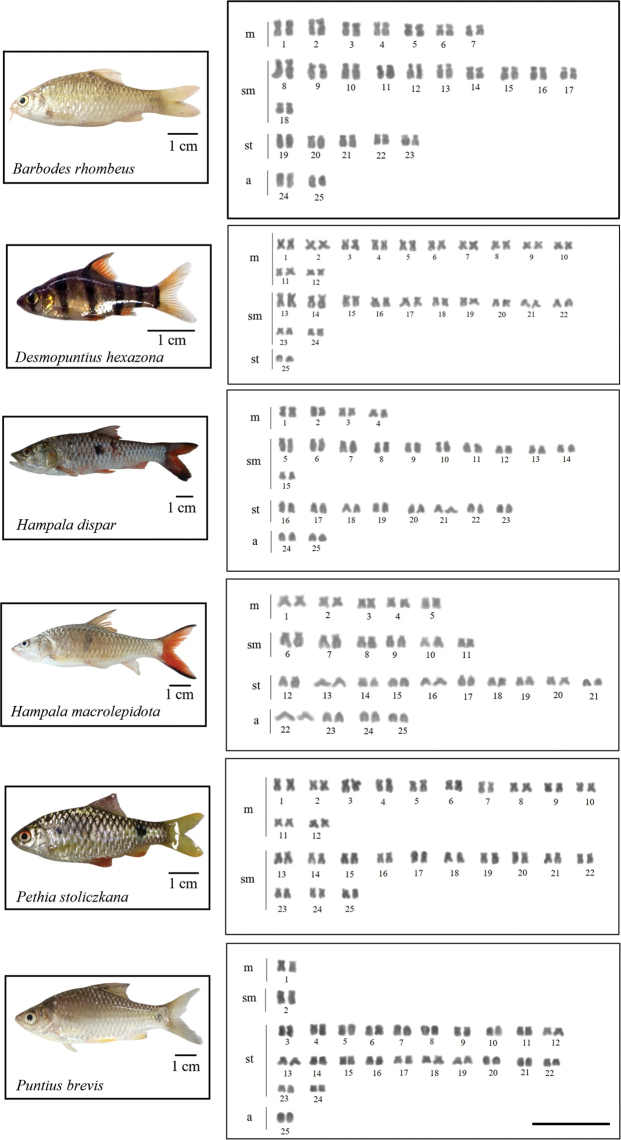
Karyotypes of Smiliogastrinae species arranged from Giemsa-stained chromosomes. Scale bar: 5 µm.

**Figure 5.**
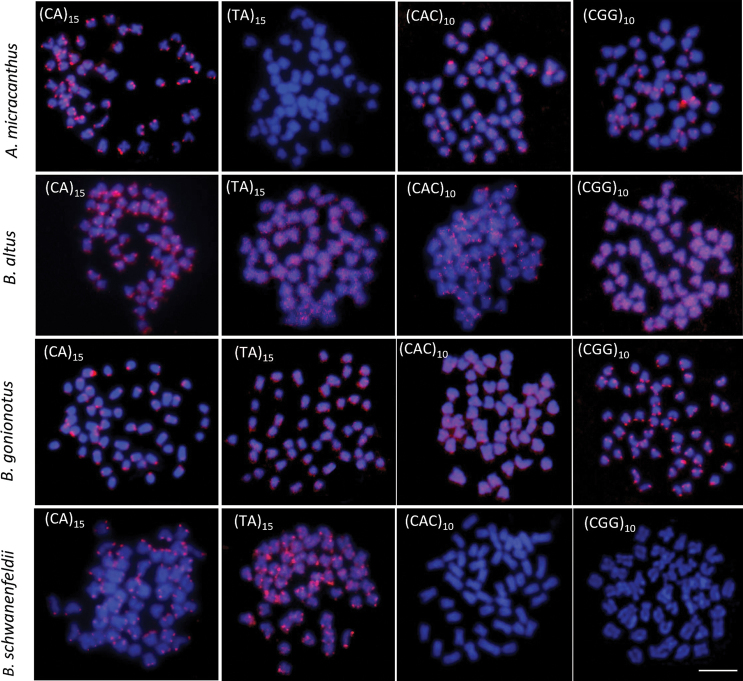
Metaphase plates of four 'Poropuntinae' species in situ hybridized with different microsatellite motifs. Scale bar: 5 μm.

**Figure 6.**
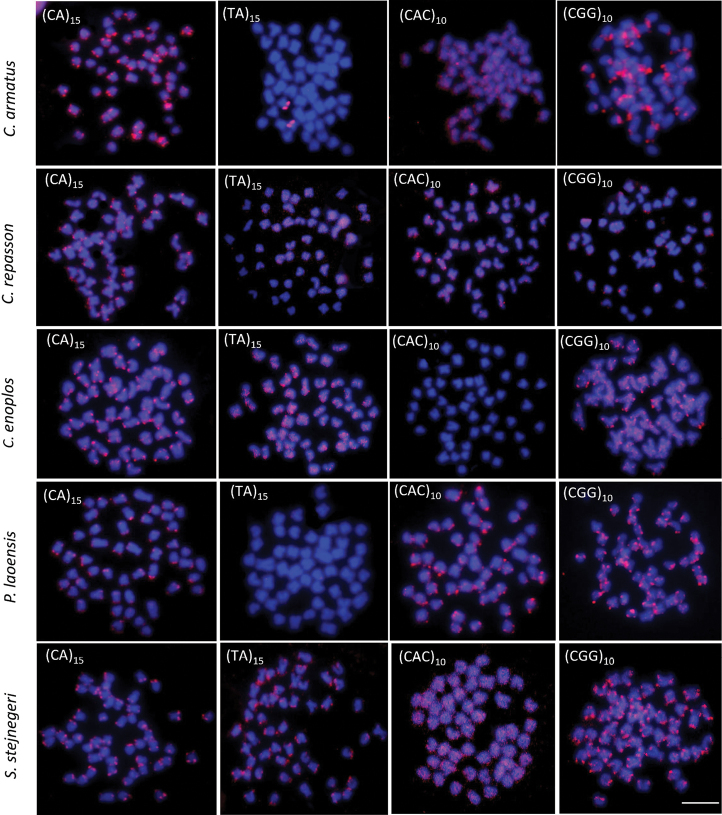
Metaphase plates of five 'Poropuntinae' species in situ hybridized with different microsatellite motifs. Scale bar: 5 μm.

**Figure 7.**
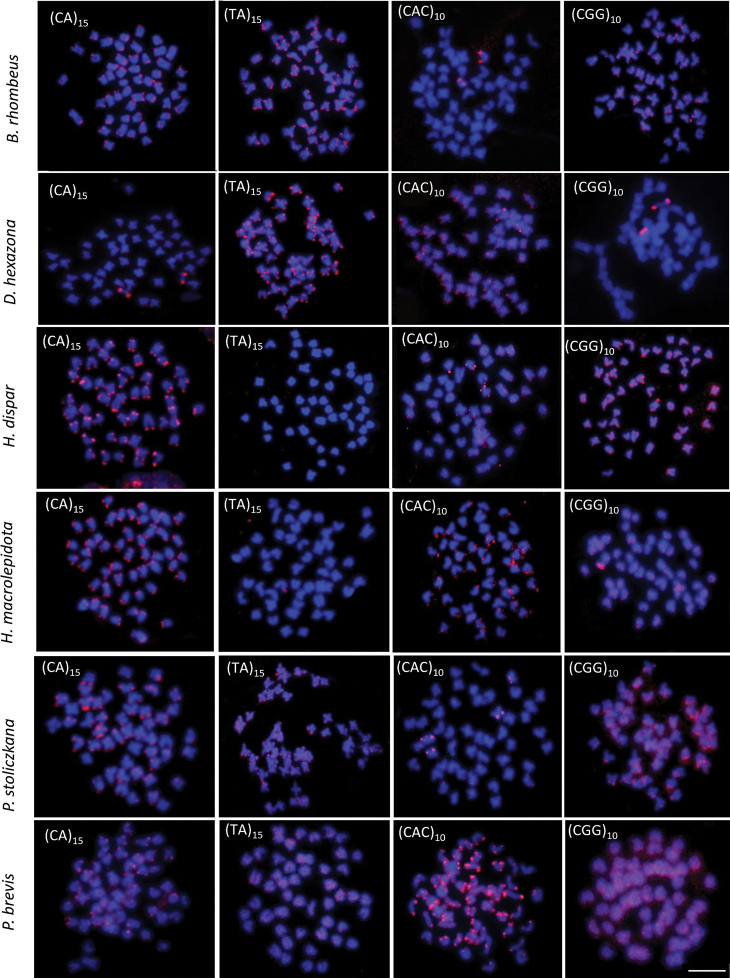
Metaphase plates of Smiliogastrinae species in situ hybridized with different microsatellite motifs. Scale bar: 5 μm.

All 15 analyzed species had a 2n = 50. Nevertheless, chromosome arm number per karyotype (fundamental number; FN) were unique to each species (see Table 2 and Figs 2–4). Four microsatellites, specifically (CA)_15_, (TA)_15_, (CAC)_10_, and (CGG)_10_, were used to hybridize onto the chromosomes of all species under investigation. The FISH results showed that the chromosomes of 14 out of the 15 species exhibited a very similar distribution of the (CA)_15_ microsatellite sequences. The (CA)_15_ probe exhibited a robust signal and selectively hybridized in the telomeric regions of all chromosomes. On the other hand, the bright signal for (CA)_15_ in *D.hexazona* was evenly spread across the telomeric regions of all chromosomes, albeit only one pair exhibited a prominent signal. The (TA)_15_ probe did not show positive hybridization signals in the chromosomes of *A.micracanthus* (Fig. 5), *P.laoensis* (Fig. 6), *H.dispar* and *H.macrolepidota* (Fig. 7). Similarly, both the (CAC)_10_ and (CGG)_10_ probes did not exhibit positive signals on the chromosomes of *B.schwanenfeldii* (Fig. 5). The (TA)_15_ probe signals were detected in the telomeric regions of chromosomes in *B.gonionotus*, *B.schwanenfeldii* (Fig. 5), *S.stejnegeri* (Fig. 6), *B.rhombeus*, *D.hexazona* and *P.stoliczkana* (Fig. 7). The species that show (TA)_15_ spread signals on chromosomes of *B.altus* (Fig. 5), *C.repasson* (Fig. 6), and *P.brevis* (Fig. 7). In addition, the chromosomes of *C.enoplos* exhibited a fairly dispersed (TA)_15_ signal throughout all chromosomes, but a strong hybridization pattern was observed in nearly all telomeric regions. However, just one specific telomeric signal was detected in a single pair of chromosomes in *C.armatus* (Fig. 6). The bulk of the examined fish samples exhibit comparable patterns of (CAC)_10_ and (CGG)_10_, which are predominantly found in telomeric regions. However, *B.gonionotus* displayed only the (CAC)_10_ signals, a scattered distribution throughout all chromosomes. Furthermore, both *B.rhombeus* and *P.stoliczkana* had a single pair of chromosomes that have a unique (CAC)_10_ signal. Similarly, the (CGG)_10_ signal was specifically distributed in the chromosomes of *D.hexazona* and *H.macrolepidota*, appearing in one and two pairs of chromosomes, respectively (Table 2; Figs 5–7).

**Table 2.** Karyotypes and distribution profiles of the microsatellite repeats in the genomes of 15 fish species from the 'Poropuntinae' and Smiliogastrinae. 2n = diploid chromosome number, FN = fundamental number (number of chromosome arms), m = metacentric, sm = submetacentric, st = subtelocentric, a = acrocentric, telomeric = high accumulation on all telomeres of all chromosomes, spread = high accumulation throughout chromosome in most/all chromosomes, specific = some accumulation on telomere of a few chromosome pairs and - = not available.

Species	2n	FN	Karyotype	Microsatellite distribution patterns			
				(CA)_15_	(TA)_15_	(CAC)_10_	(CGG)_10_
'**Poropuntinae**'							
*Amblyrhynchichthysmicracanthus*	50	96	14m+20sm+12st+4a	telomeric	–	telomeric	telomeric
*Barbonymusaltus*	50	96	20m+10sm+16st+4a	telomeric	spread	telomeric	spread
*B.gonionotus*	50	94	10m+22sm+12st+6a	telomeric	telomeric	spread	telomeric
*B.schwanenfeldii*	50	94	6m+18sm+20st+6a	telomeric	telomeric	–	–
*Cyclocheilichthysarmatus*	50	94	12m+18sm+14st+6a	telomeric	specific	spread	telomeric
*C.repasson*	50	96	12m+22sm+12st+4a	telomeric	spread	spread	telomeric
*Cyclocheilosenoplos*	50	98	14m+22sm+12st+2a	telomeric	spread	–	spread/ telomeric
*Poropuntiuslaoensis*	50	90	12m+16sm+12st+10a	telomeric	–	telomeric	telomeric
*Sikukiastejnegeri*	50	88	6m+16sm+16st+12a	telomeric	telomeric	spread	telomeric
**Smiliogastrinae**							
*Barbodesrhombeus*	50	96	14m+22sm+10st+4a	telomeric	telomeric	specific	telomeric
*Desmopuntiushexazona*	50	100	24m+24sm+2st	specific	telomeric	telomeric	specific
*Hampaladispar*	50	96	8m+22sm+16st+4a	telomeric	–	specific	spread
*H.macrolepidota*	50	92	10m+12sm+20st+8a	telomeric	–	telomeric	spread
*Pethiastoliczkana*	50	100	24m+26sm	telomeric	telomeric	specific	spread
*Puntiusbrevis*	50	98	2m+2sm+44st+2a	telomeric	spread	telomeric	spread

## ﻿Discussion

### ﻿Chromosomal features of the 15 Cyprinidae species under study

The diploid chromosome number was found to be 50 in all species studied, which supports the findings of previous cytogenetic investigations (Magtoon and Arai 1989; Donsakul et al. 2005, 2006; Seetapan 2007; Chantapan 2015; Supiwong et al. 2017; Chaiyasan et al. 2018, 2020; Buasriyot et al. 2024). Nevertheless, the 2n of *Puntiusbrevis* is not consistent with the findings of Seetapan (2007), who observed a diploid number of 48. Both lineages, ´Poropuntinae´ and Smiliogastrinae, had a significant level of conservation for the 2n chromosome count, as demonstrated by Arai (2011); Phimphan et al. (2020); Khensuwan et al. (2023a, 2024b), and the present study.

The value of 2n = 50 is found in many different species of cyprinoid fish and is consistently present throughout their evolutionary history (Arai 2011). While 2n remains conserved, the FN value varies widely. When comparing previous and present investigation, the FN in the present study differs from most of the previous studies, except for the report by Buasriyot et al. (2024). The FN varied across different species within the Smiliogastrinae subfamily, ranging from 56 in *Puntiusbrevis* (Seetapan 2007) to 100 in *D.hexazona* (present study) and *Pethiastoliczkana* (Buasriyot et al. 2024; present study). Similarly, within the ´Poropuntinae´ subfamily, the FN ranged from 66 in *B.gonionotus* (Seetapan 2007) to 98 in *Cyclocheilosenoplos* (Buasriyot et al. 2024; present study).

The karyotypes of almost 15 fish species had all four types of chromosomes (m, sm, st, and a) (Figs 2–4), except for *D.hexazona* and *P.stoliczkana*, which contain three types (m, sm, and st) and two types (m and sm) of chromosomes, respectively (Fig. 4). The inconsistencies may arise due to variations within populations or species, as well as inaccurate identification of one species as another, which can be attributed to the complexity of species (Phimphan et al. 2020). According to Demarais’s (1992) findings, chromosomal evolution in these cyprinids can occur among populations separated by a geographic barrier. This evolution can result in the reorganization of chromosome types through pericentric inversion (or other mechanisms causing centromeric shifts), resulting in the observed variety. Based on the FN and karyotypic structure, it appears that a higher FN and a greater number of bi-armed chromosomes are indicative of a more apomorphic character compared to a lower FN and a greater number of mono-armed chromosomes. Therefore, the karyotypes of *D.hexazona* and *P.stoliczkana* are expected to exhibit more apomorphic characteristics. None of the fifteen species examined had individuals with heteromorphic sex chromosomes in both males and females. Arai (2011) states that several animals within this family display a consistent pattern of undifferentiated sex chromosomes.

### ﻿Microsatellite distribution patterns

Microsatellites are typically located in the telomeric and centromeric regions of autosomal and sex chromosomes in fish genomes, often linked with other repetitive DNA sequences (Cioffi and Bertollo 2012). The findings of our investigation indicate a significant build-up of the microsatellite (CA)_15_ in the genomes of nearly all species, particularly in telomeric areas. This observation aligns with the research conducted by Cioffi et al. (2011), Ditcharoen et al. (2020), and Khensuwan et al. (2023a, 2024). Except for the *D.hexazona*, just a single pair of (CA)_15_ signal was observed. The observed pattern applies to the cyprinid species investigated in this study, since they exhibit a consistent distribution of (CA)_15_ motifs in the telomeric region of all chromosomes. The species present in the group include *Puntigruspartipentazona* Fowler, 1934 (Phimphan et al. 2020), other species belonging to the subfamily ‘Poropuntiinae’ (Khensuwan et al. 2023a), and several species from the subfamily Labeoninae, such as *Epalzeorhynchosfrenatum* Fowler, 1934 (Phimphan et al. 2020), *E.bicolor* Smith, 1931, *E.munense* Smith, 1934, *Henicorhynchussiamensis* Sauvage, 1881, and *Thynnichthysthynnoides* Bleeker, 1852 (Khensuwan et al. 2024). Recent studies indicate that the distribution and quantity of microsatellite sequences on chromosomes can differ among closely related fish species. This has been observed in channid fish, bagrid catfish, and silurid catfish, as described by Supiwong et al. (2014), Cioffi et al. (2015), and Ditcharoen et al. (2020).

The distribution of microsatellite motifs in fish genomes may exhibit a bias towards specific noncoding regions, but it may also correlate with the distribution of rDNAs across chromosomes (e.g. Sassi et al. 2019; Ditcharoen et al. 2020; Mingkwan et al. 2023). The distribution pattern of (CA)_15_ in *D.hexazona* resembled that of *Trichopsisschalleri* Ladiges, 1962 and *T.vittata* Cuvier, 1831 in a previous work conducted by Mingkwan et al. 2023. It exhibited a substantial accumulation in the centromeric region of certain chromosome pairs. However, it is distinct from the genus *Osteochilus* (Saenjundaeng et al. 2020), *T.pumila* Arnold, 1936 (Mingkwan et al. 2023), *Belontiahasselti* Cuvier, 1831 (Chaiyasan et al. 2021), *Mystus* species (Yeesin 2021), the Thai pufferfish *Pao cochinchinensis* Steindachner, 1866 (Pissaparn et al. 2020), and silurid species (Ditcharoen et al. 2020), as it contains (CA)_15_ sequences throughout its entire karyotype. Despite the presence of repetitive DNA in the telomeric regions, which are mainly composed of the (TA)_15_, (CAC)_10_, and (CGG)_10_ motifs, different species display highly varied hybridization patterns for the same microsatellite. The motifs (TA)_15_, (CAC)_10,_ and (CGG)_10_ exhibit four distinct patterns of distribution: 1. High accumulation in all telomeric regions of most or all chromosomes in multiple species; 2. High accumulation spread throughout the entire genomes of multiple species; 3. Some accumulation in telomeric sites of a few chromosomes, such as the (TA)_15_ repeats in *C.armatus*, the (CAC)_10_ repeats in *B.rhombeus*, *H.dispar*, and *P.stoliczkana*, and the (CGG)_10_ repeats in *D.hexazona*. 4. Non-clustered organization in certain species, such as the (TA)_15_ repeats in *A.micracanthus*, *H.dispar*, *H.macrolepidota*, and *P.laoensis*, the (CAC)_10_ repeats in *B.schwanenfeldii* and *C.enoplos*, and the (CGG)_10_ repeats in *B.schwanenfeldii*.

Three patterns, excluding those where positive FISH signals were not encountered, are in agreement with earlier studies conducted on Labeoninae (Saenjundaeng et al. 2020: Khensuwan et al. 2024), 'Poropuntiinae' (Khensuwan et al. 2023a, 2023b), and Smiliogastrinae (Phimphan et al. 2020). The karyotypes displayed species-specific microsatellite distribution patterns. The results suggest that the (CAC)_10_ and (CGG)_10_ repetitions could serve as a marker specific to the genus *Barbonymus*, while the (TA)_15_ repeats could serve as a marker distinct to the genus *Cyclocheilichthys*. Furthermore, the (TA)_15_ and (CAC)_10_ repeats are appropriate to serve as species-specific identifiers for the genus *Hampala*.

Repetitive DNA has been discovered in heterochromatic regions of fishes, such as telomeres, centromeres, or portions of sex or B chromosomes, according to multiple studies (Cioffi and Bertollo 2012; Supiwong et al. 2014; Ditcharoen et al. 2020; Khensuwan et al. 2023). Nevertheless, the majority of microsatellite sequences found in the genus *Osteochilus* are dispersed throughout the chromosomes and do not exhibit any particular association with heterochromatic regions (Saenjundaeng et al. 2020). In addition, microsatellites can also be located in regions outside of the centromere, often near or within genes (Getlekha et al. 2016b). The buildup of repetitive DNA is recognized as the main driver for karyotype diversification associated with speciation (Dernberg et al. 1996; Maneechot et al. 2016; Ditcharoen et al. 2020). Repetitive DNA mapping, specifically of microsatellites and ribosomal DNA classes, can be used to study and estimate evolutionary karyotype changes in certain fish species (Getlekha et al. 2016a; Maneechot et al. 2016; Ditcharoen et al. 2019; Ditcharoen et al. 2020; Saenjundaeng et al. 2020; Chaiyasan et al. 2021; Khensuwan et al. 2023a, 2024). Indeed, repetitive DNA has been observed to play a significant role in the evolution of the genome in several fish species (Cioffi and Bertollo 2012; Yano et al. 2014; Moraes et al. 2019; Saenjundaeng et al. 2020; Khensuwan et al. 2023a, 2024).

Recent research has identified functional microsatellites that influence an individual’s physical attributes (Padeken et al. 2015). The microsatellite sequences discovered in the present study were located in a manner comparable to retroelements within the same species (Suntronpong et al. 2017). Some signals were concentrated in the terminal and centromeric regions, while others were dispersed across the chromosomes (Schneider et al. 2013). The microsatellites (TA)_15_, (CAC)_10,_ and (CGG)_10_ are distributed across the chromosome, exhibiting dispersed patterns and distinct markings in the telomeric regions of the majority of them.

Some authors (Cioffi et al. 2012; Garrido Ramos 2017; Moraes et al. 2017; Saenjundaeng et al. 2020; Khensuwan et al. 2023a, 2024) argue that repetitive elements serve as indicators of evolutionary processes, facilitating the detection of recent karyotype modifications, including chromosome rearrangements, distinct microsatellite distribution patterns, and multiple rDNA loci. The comparative cytogenetic mapping results presented here not only enhance our comprehension of the genome of this fish family but also provide novel insights into the structure and organization of the repetitive DNA region in the Systomini genomes.

## ﻿Conclusions

This research applied conventional and molecular cytogenetics to examine the karyotypic organization and microsatellite distribution in 15 fish species from the subfamilies Poropuntiinae and Smiliogastrinae. All studied species have the same diploid chromosome number, 2n = 50; however, they have different fundamental numbers and unique karyotype arrangements. Microsatellites (CA)_15_, (TA)_15_, (CAC)_10_, and (CGG)_10_ exhibit distinct distribution patterns, characterized by a high accumulation in the telomeric region of some or all chromosomes, as well as a species-specific widespread distribution across the genome. These results indicate that microsatellites can be valuable genetic markers that differentiate inside genera with similar morphology.

## ﻿Author contributions

**Conceptualization**: Phichaya Buasriyot, Weerayuth Supiwong, Nawarat Muanglen, Sampan Tongnunui, Alongklod Tanomtong, Marcelo de Bello Cioffi. **Data curation**: Phichaya Buasriyot, Nawarat Muanglen, Weerayuth Supiwong, Petr Ráb, Thomas Liehr, Marcelo de Bello Cioffi. **Formal analysis**: Phichaya Buasriyot, Weerayuth Supiwong, Petr Ráb, Thomas Liehr, Marcelo de Bello Cioffi. **Funding acquisition**: Weerayuth Supiwong, Nawarat Muanglen, Kriengkrai Seetapan, Alongklod Tanomtong, Thomas Liehr. **Investigation**: Nuttasuda Donbundit, Nawarat Muanglen, Sampan Tongnunui, Pasakorn Saenjundaeng, Sitthisak Juntharat, Kriengkrai Seetapan. **Methodology**: Phichaya Buasriyot, Sukhonthip Ditcharoen, Pasakorn Saenjundaeng, Weera Thongnetr, Nuttasuda Donbundit, Sitthisak Juntharat, Sampan Tongnunui, Kriengkrai Seetapan, Phonluang Chumpol, Weerayuth Supiwong, Petr Ráb, Nawarat Muanglen, Thomas Liehr, Marcelo de Bello Cioffi. **Software**: Thomas Liehr, Marcelo de Bello Cioffi, Weerayuth Supiwong, Alongklod Tanomtong. **Project administration**: Weerayuth Supiwong, Nawarat Muanglen, Alongklod Tanomtong, Thomas Liehr. **Resources**: Weerayuth Supiwong, Alongklod Tanomtong, Nawarat Muanglen, Thomas Liehr, Marcelo de Bello Cioffi. **Supervision**: Weerayuth Supiwong, Sampan Tongnunui, Satit Arunsang, Nawarat Muanglen, Alongklod Tanomtong, Thomas Liehr, Marcelo de Bello Cioffi, Petr Ráb. **Validation**: Petr Ráb, Marcelo de Bello Cioffi, Weerayuth Supiwong, Thomas Liehr, Alongklod Tanomtong. **Visualization**: Phichaya Buasriyot, Weerayuth Supiwong, Nawarat Muanglen, Alongklod Tanomtong. **Writing – original draft**: Phichaya Buasriyot, Nuttasuda Donbundit, Sukhonthip Ditcharoen, Nawarat Muanglen, Alongklod Tanomtong, Thomas Liehr, Weerayuth Supiwong. **Writing – review & editing**: Phichaya Buasriyot, Marcelo de Bello Cioffi, Petr Ráb, Thomas Liehr, Weerayuth Supiwong, Alongklod Tanomtong, Nawarat Muanglen, Sampan Tongnunui.

## ﻿ORCID

**Phichaya Buasriyot** https://orcid.org/0000-0003-0821-7629

**Weerayuth Supiwong** https://orcid.org/0000-0002-1670-3224

**Nawarat Muanglen** https://orcid.org/0000-0003-4529-4252

**Nattasuda Donbundit** https://orcid.org/0000-0002-3074-9288

**Sukhonthip Ditcharoen** https://orcid.org/0009-0009-8753-725X

**Phonluang Chumpol** https://orcid.org/0009-0002-7896-2210

**Pasakorn Saenjundaeng** https://orcid.org/0009-0005-9337-2683

**Sampan Tongnunui** https://orcid.org/0009-0002-4899-908X

**Sathit Arunsang** https://orcid.org/0009-0005-7574-5757

**Weera Thongnetr** https://orcid.org/0009-0000-2598-3144

**Sitthisak Juntharat** https://orcid.org/0009-0006-0376-0808

**Kriengkrai Seetapan** https://orcid.org/0000-0003-0900-6890

**Thomas Liehr** https://orcid.org/0000-0003-1672-3054

**Marcelo B. Cioffi** https://orcid.org/0000-0003-4340-1464

**Petr Rab** https://orcid.org/0000-0002-1830-3523

**Alongklod Tanomtong** https://orcid.org/0000-0002-8466-3594
